# Prevalence of early childhood caries in Bhubaneswar, Odisha, India

**DOI:** 10.6026/973206300210087

**Published:** 2025-01-31

**Authors:** Kanika Singh Dhull, Brahmananda Dutta, Mukesh Soni, Aditi Burman, Abhinav Chand Singhal, Shreya Shrinivas, Aditi Gupta, Pratik Surana

**Affiliations:** 1 Department of Pedodontics & Preventive Dentistry, Kalinga Institute of Dental Sciences, Kalinga Institute of Industrial Technology (Deemed to be University), Bhubaneswar, Odisha, India; 2 Department of Prosthodontics, Crown and Bridge and Implantology, Government College of Dentistry, Indore, Madhya Pradesh, India; 3 Department of Pediatric and Preventive Dentistry, SCB Dental College and Hospital, Cuttack, Odisha, India; 4 Department of Pedodontics and Preventive Dentistry, Rungta College of Dental Sciences and Research, Bhilai, Chhattisgarh, India; 5 Aids Institute, Health research Inc., Albany, New York, USA; 6 Department of Pedodontics and Preventive Dentistry, Maitri College of Dentistry and Research Centre, Durg, Chhattisgarh, India

**Keywords:** Early childhood caries, early childhood caries, prevalence

## Abstract

Early Childhood Caries (ECC) poses a major challenge to young children's oral health worldwide. Therefore, it is of interest to explore the prevalence of early childhood (between 3-6 years) caries in Bhubaneswar, Odisha. An oral examination of 460 children, chosen using a random sampling, revealed a 54.78% prevalence of early childhood caries. Data underscore the need for community-based prevention, public health education targeting parents and caregivers through integrating oral health promotion into primary healthcare to combat early childhood caries effectively in Bhubaneswar.

## Background:

Dental caries is among the most widespread conditions, capable of developing at any age once teeth emerge [1]. Dental caries, commonly known as tooth decay, specifically in the primary dentition, is a significant public health concern [2]. In pediatric populations, dental caries exhibits unique characteristics and poses specific challenges. Notably, it is the most prevalent chronic condition in childhood, occurring at rates five times higher than asthma and seven times higher than hay fever [3, 4]. Early childhood caries patients frequently are underweight, iron insufficiency and delayed growth because of discomfort that makes feeding difficult [5]. Since 1962, various terminologies have described caries in young children, such as nursing bottle syndrome, bottle mouth caries and several others. To consolidate these terms and emphasize the multifactorial nature of the condition, the term early childhood caries has been advocated for all forms of caries in infants and preschool-aged children [1, 6 and 7]. The prevalence of caries in preschool-aged children varies greatly, according to epidemiological statistics, with estimates ranging from 3% to 85%. These differences are closely associated with both socioeconomic and ethnic characteristics [8, 9, 10-11]. Recent evidence from Scotland has shown a decrease in caries prevalence among 3-year-olds, which is likely attributable to local preventative programs. Therefore, it is of interest to report the prevalence of early childhood caries in Bhubaneswar, Odisha, India.

## Materials and Methods:

This pilot study was conducted in Bhubaneswar; Odisha, India, targeting children aged 3 to 6 years who were attending various primary schools. The primary aim was to assess the prevalence of early childhood caries within this demographic. The research sample comprised 460 children, chosen using a stratified random sampling method to ensure a representative distribution across different socioeconomic backgrounds, with the sample size calculated to ensure statistically significant results based on prior prevalence studies. To be included in the study, children had to be between 3 and 6 years old, attending the selected primary schools and have parental or guardian consent. Exclusion criteria included children with systemic diseases, those on medications affecting salivary flow, or those who had undergone dental treatments that might interfere with caries detection. The study received ethical permission from IEC of Kalinga Institute of Dental Sciences, Bhubaneswar, Odisha (Ref. no KIIT/KIMS/IEC/1771/2024). Permission for participation was gathered from the parents or guardians of all the children involved. Data collection involved clinical examinations conducted by a trained dentist under natural lighting conditions, using a dental mirror and probe, following the World Health Organization (WHO) guidelines for diagnosing early childhood caries. Early childhood caries was defined by the presence of one or more decayed, missing (due to caries), or filled tooth surfaces in any primary tooth. The severity of dental caries was assessed using the DMFT index, which records the number of decayed, missing and filled teeth. Data were collected and statistically analyzed.

## Results:

Among the 460 children examined (244 girls and 216 boys) (Table 1), 252 were diagnosed with early childhood caries, reflecting an overall prevalence of 54.78%. Boys exhibited a higher prevalence rate at 59.25%, compared to girls at 50.81%. Nonetheless, the data did not reveal any statistically significant link between the occurrence of early childhood caries and the child's sex (Table 2).

## Discussion:

Early childhood caries also referred to as "baby bottle tooth decay" or maxillary anterior caries remains a pervasive challenge in both industrialized and developing nations. Even though dental professionals have a thorough understanding of the etiological elements that lead to early childhood caries (ECC), early childhood caries is still the most prevalent and misdiagnosed oral health problem in children. Because the American Academy of Pediatric Dentistry (AAPD) specifies exact age-restricted requirements, the name "early childhood caries" is preferred. Early childhood caries is characterised as a multifactorial, chronic, infectious illness that is caused by cariogenic bacteria, fermentable carbohydrates in food, susceptible dentition and temporal variables [2, 3]. When early childhood caries first appears in babies, it usually shows up as white demineralised lesions in the maxillary anterior teeth's cervical regions. Preventive interventions must be developed and put into action for pre-schoolers who have been recognised as being at risk. Many risk factors, such as age, gender, ethnicity, food habits and dental hygiene routines, influence the prevalence and incidence of caries in a community. Developing effective preventive and treatment measures requires a full understanding of the prevalence of caries and the risk factors connected with it. Thus, it is always necessary to assess the prevalence of caries and the associated risk factors [4, 6]. The study included 460 healthy preschool children aged 3 to 6 years. Children who were uncooperative or had any medical condition were omitted from the study. Dental caries were recorded using the decayed, missing, and filled permanent teeth (DMFT) index and parents were interviewed about the associated risk factors for early childhood caries. The prevalence of early childhood caries among children aged 3 to 6 in this study was 54.78%, which is lower than the 67% reported in previous studies conducted in Nellore [12]. However, it is higher than the prevalence reported in various other parts of India, including Bengaluru (27.5%) [13], West Godavari District Andhra Pradesh (41.9%) [14], Salem, Tamil Nadu (16%) [15] and Haryana (33%) [16]. Similarly Ganesh *et al.* in 2019 analysed various Indian researches and revealed the overall prevalence of early childhood caries in India to be 49.6% which was lesser than current research [17]. In contrast to some of the lower worldwide indices, the local prevalence of early childhood caries is higher, which emphasises the urgent need for targeted public health measures. It seems that Bhubaneswar needs specific dental health education and preventive measures designed for its particular demographic and socioeconomic environment [13, 14, 15-16]. Dental health initiatives that are centred in schools could be extremely important in raising awareness and encouraging good oral hygiene habits in young children and their families [17]. Preventive dental health strategies, such as community fluoride programs, regular dental check-ups and educational initiatives for caregivers, could substantially mitigate the risks of early childhood caries. Considering the age group studied, educational programs should be designed to engage both the children and their caregivers, emphasizing the importance of early dental care and proper feeding practices [18]. The results support the need for on-going study and observation to monitor early childhood caries trends and assess the success of adopted tactics. Longitudinal studies have the potential to provide significant insights into the long-term impacts of early interventions, which could aid in the improvement of public health policy. Furthermore, a deeper investigation into ethnic relationships may reveal particular community requirements and cultural norms influencing oral health.

## Conclusion:

Substantial contribution to the current corpus of knowledge regarding early childhood caries, advocating for intensified efforts in prevention and education is essential. It will be essential to address the highlighted socio-economic and ethnic determinants in order to lower the prevalence of early childhood caries in Bhubaneswar. Future programs should integrate targeted, on-going research with public health measures to address early childhood caries and promote healthier generations.

## Figures and Tables

**Table 1:** Demographic distribution of participants as per gender

**Gender**	**Participants**
Boys	216 (47%)
Girls	244 (53%)
Total	460 (100%)

**Table 2:** Prevalence of ECC

**Gender**	**Number**	**Participants with ECC**	**Participants Without ECC**	**Mean DMFT**
Boys	216 (47%)	128 (59.25%)	88	
Girls	244 (53%)	124 (50.81%)	120	4.32 ± 0.65
Total	460	252 (54.78%)		
